# *QuickStats:* Prevalence* of Past or Present Infection with Hepatitis B Virus^†^ Among Adults Aged ≥18 Years, by Race and Hispanic Origin — National Health and Nutrition Examination Survey, 1999–2018

**DOI:** 10.15585/mmwr.mm6935a8

**Published:** 2020-09-04

**Figure:**
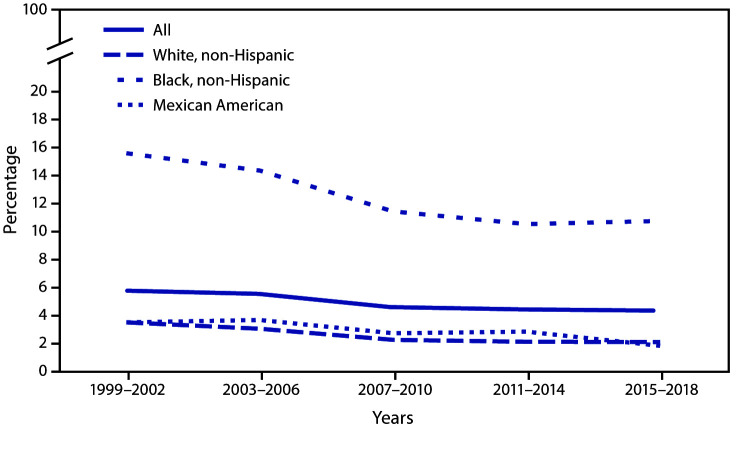
The prevalence of past or present infection with hepatitis B virus among adults aged ≥18 years declined from 5.7% in 1999–2002 to 4.3% in 2015–2018. A decline among non-Hispanic White (3.5% to 2.1%), non-Hispanic Black (15.6% to 10.8%), and Mexican American (3.5% to 1.8%) adults also occurred over the same period. Prevalence was higher among non-Hispanic Black adults than among both non-Hispanic White and Mexican American adults for all periods.

**Sources:** Kruszon-Moran D, Paulose-Ram R, Martin CB, Barker L, McQuillan G. Prevalence and trends in hepatitis B virus infection in the United States, 2015–2018. NCHS Data Brief, no 361. https://www.cdc.gov/nchs/products/databriefs/db361.htm; National Center for Health Statistics, National Health and Nutrition Examination Survey, 1999–2002 to 2015-2018. https://www.cdc.gov/nchs/nhanes/index.htm.

